# Ashwagandha-loaded nanocapsules improved the behavioral alterations, and blocked MAPK and induced Nrf2 signaling pathways in a hepatic encephalopathy rat model

**DOI:** 10.1007/s13346-022-01181-y

**Published:** 2022-06-07

**Authors:** Heba M. A. Khalil, Islam A. Khalil, Asmaa K. Al-Mokaddem, Marwa Hassan, Riham A. El-Shiekh, Hesham A. Eliwa, Azza M. Tawfek, Walaa H. El-Maadawy

**Affiliations:** 1 Department of Veterinary Hygiene and Management, Faculty of Veterinary Medicine, Cairo University, Giza, 12211 Egypt; 2 Department of Pharmaceutics, College of Pharmaceutical Sciences and Drug Manufacturing, Misr University of Science and Technology (MUST), 6th of October, Giza, 12582 Egypt; 3 Department of Pathology, Faculty of Veterinary Medicine, Cairo University, Giza Square, Giza, 12211 Egypt; 4 Department of Immunology, Theodor Bilharz Research Institute, Kornaish El Nile, Warrak El-Hadar, Imbaba, P.O. 30, Giza, 12411 Egypt; 5 Department of Pharmacognosy, Faculty of Pharmacy, Cairo University, Kasr el Aini st., Cairo, 11562 Egypt; 6 Department of Pharmacology and Toxicology, College of Pharmacy and Drug Manufacturing, Misr University of Science and Technology (MUST), 6th October, Giza, 12566 Egypt; 7 Department of Clinical Pathology, Faculty of Veterinary Medicine, Cairo University, Giza, 12211 Egypt; 8 Department of Pharmacology, Theodor Bilharz Research Institute, Kornaish El Nile, Warrak El-Hadar, Imbaba, P.O. 30, Giza, 12411 Egypt

**Keywords:** Ashwagandha, Bipolymeric nanocapsules, Hepatic encephalopathy, Cognition, Nrf2 pathway, MAPK pathway

## Abstract

Ashwagandha (ASH), a vital herb in Ayurvedic medicine, demonstrated potent preclinical hepato- and neuroprotective effects. However, its efficacy is limited due to low oral bioavailability. Accordingly, we encapsulated ASH extract in chitosan–alginate bipolymeric nanocapsules (ASH-BPNCs) to enhance its physical stability and therapeutic effectiveness in the gastrointestinal tract. ASH-BPNC was prepared by emulsification followed by sonication. The NCs showed small particle size (< 220 nm), zeta-potential of 25.2 mV, relatively high entrapment efficiency (79%), physical stability at acidic and neutral pH, and in vitro release profile that extended over 48 h. ASH-BPNC was then investigated in a thioacetamide-induced hepatic encephalopathy (HE) rat model. Compared with free ASH, ASH-BPNC improved survival, neurological score, general motor activity, and cognitive task-performance. ASH-BPNC restored ALT, AST and ammonia serum levels, and maintained hepatic and brain architecture. ASH-BPNC also restored GSH, MDA, and glutathione synthetase levels, and Nrf2 and MAPK signaling pathways in liver and brain tissues. Moreover, ASH-BPNC downregulated hepatic NF-κB immunohistochemical expression. Moreover, the in vivo biodistribution studies demonstrated that most of the administered ASH-BPNC is accumulated in the brain and hepatic tissues. In conclusion, chitosan–alginate BPNCs enhanced the hepatoprotective and neuroprotective effects of ASH, thus providing a promising therapeutic approach for HE.

**Graphical abstract:**

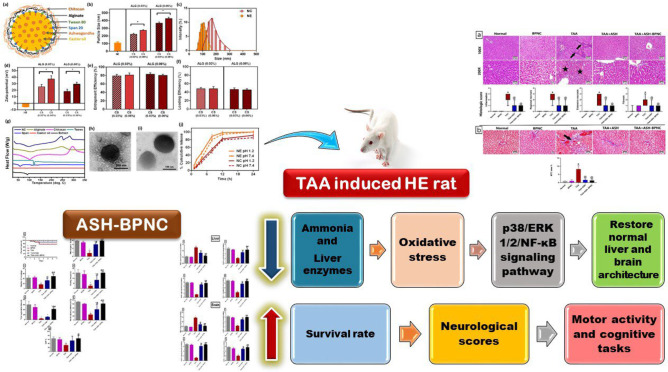

## Introduction

Hepatic encephalopathy (HE) is a severe recurrent syndrome resulting from chronic liver diseases and acute liver failure (ALF). HE includes a broad spectrum of neuropsychiatric abnormalities, ranging from mild cognitive impairment to marked disorientation, confusion, and coma. It has an enormous clinical and economic burden and contributes to increased morbidity and mortality rates [1]. The etiology of HE is complex and multifactorial, but it is commonly agreed that an increase in systemic ammonia concentrations plays a chief role in its pathophysiology, in addition to neuroinflammation and oxidative stress [2].

Despite increased understanding of the pathophysiology of HE, targeting hyperammonemia remains the cornerstone of treatment [1]. The initial therapy for HE is currently a combination of nonabsorbable disaccharides such as lactulose and nonabsorbable antibiotics, including rifaximin [3]. Other therapeutic options include probiotics, L-ornithine L-aspartate, and branched-chain amino acids. However, there are no evidence-based results to suggest these agents have an influential role on HE [4]. Evolving evidence supports the proposition that targeting multiple pathways implicated in HE is a promising approach for HE treatment [5].

*Withania somnifera* (L.) Dunal, family Solanaceae, generally known as ashwagandha (ASH), is a perennial medicinal herb rich in steroidal lactones, saponins, alkaloids, flavonoids, and withanolides [6]. ASH has been applied to treat memory-related conditions and improve learning ability and memory capacity. Preclinical studies indicated that ASH promotes cognitive function and memory enhancement, as well as prevents cognitive deficits and neurodegeneration [7–9]. ASH and withanolides are reported to possess potent antioxidant [10, 11] and hepatoprotective properties [12] owing to the activation of nuclear factor erythroid 2-related factor 2 (Nrf2) and deactivation of mitogen-activated protein kinase (MAPK) signaling pathways [13]. Interestingly, we have recently demonstrated that ASH root extract exhibited hepatoprotective and neuroprotective effects against thioacetamide (TAA)-induced HE in rats [14]. Specifically, it ameliorated cognitive deficits, hepatotoxicity indices, systemic ammonia levels, and brain and hepatic histopathological alterations and had potent anti-inflammatory and antioxidant activities mediated via the Nrf2 and MAPK/ nuclear factor (NF)-κB pathways. Nrf2 is an important player in the maintenance of cellular homeostasis. Under normal conditions, Nrf2 is located in cytoplasm bound to the actin-binding protein Kelch-like ECH-associated protein 1 (Keap1). Under oxidative stress conditions, Nrf2 dissociates from Keap1 complex and translocate to the nucleus, leading to the transcription of ARE and its responsive genes, including hemeoxygenase-1 (HO-1), glutathione-S-transferase (GST), NAD(P)H quinone oxidoreductase 1 (NQO1), superoxide dismutase (SOD), and glutamate-cysteine ligase catalytic subunit (GCLC) [15]. Several studies documented the interrelation between Nrf2 and various liver diseases [15] and have recently pinpointed its role in HE [16–19]. Additionally, the MAPK pathway regulates several cellular activities, including proliferation, apoptosis, inflammation, and innate immunity. MAPK pathway plays a significant role in the pathogenesis of several diseases, including hepatic and neurodegenerative disorders and cancer. MAPK family includes c-Jun NH2-terminal kinase (JNK), p38 MAPK, and extracellular signal-regulated kinase (ERK) [20]. Recently, its activation has been reported to contribute mainly to the pathogenesis of HE [21].

Although ASH exhibits rapid oral absorption [22], withaferin A, one of its main bioactive compounds, demonstrates intense first-pass metabolism and is therefore considered a chief barrier in achieving good oral bioavailability in both rats and humans [23], thereby limiting its therapeutic effectiveness [24].

Nanomedicine is a promising tool in the treatment of several chronic diseases, including hepatic [25] and neurological disorders [26], and specifically HE [27]. Several drug delivery systems can overcome the physicochemical and pharmacokinetic limitations of phytopharmaceuticals, including both isolated compounds and purified extracts, by enhancing their controlled release, biodistribution, stability, and efficacy [28]. Nanoemulsion (NE) is a thermodynamically stable drug delivery system widely used to enhance the oral bioavailability of poorly soluble drugs. This system can promote gastric absorption due to its high solubilization capacity and permeation properties, thereby enhancing overall oral absorption [29]. To further increase the stability of NE in the gastrointestinal tract (GIT), an external polymeric shell, known as nanocapsules (NCs), is usually added [30]. For these shell layers, naturally occurring polymers are intensively investigated because of their biocompatibility, biodegradability, and safety in humans. Among these polymers alginate and chitosan have gained significant attention [31]. Alginate is a negatively charged polysaccharide consisting of D-mannuronic acid and L-guluronic acid blocks, which form a polymeric chain. It is insoluble in an acidic medium and soluble at higher pH, which provides a pH-responsive effect of the polymer. Chitosan is a positively charged polysaccharide consisting of D-glucosamine and N-acetyl-D-glucosamine with reactive amino and hydroxyl groups. In contrast to alginate, it is soluble in an acidic medium and insoluble at a higher pH [32]. Therefore, chitosan and alginate polyelectrolyte complexes are widely used in food and drug delivery [33].

In this study, a chitosan–alginate bipolymeric NC loaded with ASH extract (ASH-BPNC) was formulated to improve the physical stability of ASH in the GIT environment and to enhance its therapeutic efficacy in a thioacetamide (TAA)-induced rat model of HE.

## Materials and methods

### Plant material and extract

ASH was purchased from the herbal store Haraz (Cairo, Egypt) in 2019 and authenticated by Dr. Mohamed El-Gibali, Senior Botanist at Orman Botanic Garden. A voucher specimen was deposited in the Herbarium of Pharmacognosy Department, Faculty of Pharmacy, Cairo University (No. 2019–7-20). The powder (1 kg) was exhaustively extracted with distilled water (3 × 2.5 L) using an Ultra-Turrax^®^ T25 homogenizer (Janke & Kunkel IKA-Lab., Staufen, Germany) for 20 min at approximately 60 °C. The aqueous extract was then freeze-dried for the nanopreparations and biological assays (yield:9% w/w relative to dry plant).

### Preparation of ASH-BPNC

Bipolymeric NCs were prepared in two steps according to a previously reported method with some modifications [34]. The first step, emulsification, was used to prepare oil in water NE. For the oily phase, 30 mL ASH extract was mixed with 19 mL castor oil (Acros Organics, USA) and 1 g Span 20 (Acros Organics, USA) using a homogenizer (GLH 850, Omni Inc., USA) at 5000 rpm for 15 min. The oily phase was added gradually to a 100 mL solution of Tween 80 (1% w/v, Acros Organics, USA) using a homogenizer at 10,000 rpm for 45 min and then sonicated using a probe sonicator (Model LC 60/H, Elma, Germany) at 40% amplitude for 15 min with 5 s on/off and temperature adjusted to 15 °C.

In the second step, ionic gelation, a 100-mL alginate solution (0.03 or 0.06% w/v, Sigma-Aldrich, USA) was added gradually to ASH extract NE while stirring for 30 min. A 20-mL calcium chloride solution (0.06% w/v, Acros Organics, USA) was gradually added and stirred for 30 min. Finally, a 20-mL chitosan solution (0.03% or 0.06% w/v, Sigma-Aldrich, USA) in 1% acetic acid (pH 4–4.5, Acros Organics, USA) was gradually added with stirring for 30 min. The obtained NC suspension was sonicated to unify the particle size using a probe sonicator at 40% amplitude for 5 min with 5 s on/off and temperature adjusted to 15 °C. The preparation was stored at 4 °C. All samples were prepared using deionized distilled water.

### Characterization of ASH-BPNC

The mean particle size, polydispersity index, and zeta-potential were measured using dynamic light scattering (Malvern Zetasizer Nano ZS, Malvern Instruments, Malvern, UK) in deionized water at 25 °C. Surface morphology was imaged using high-resolution-transmission electron microscopy (TEM) after staining with a 1% aqueous solution of phosphotungstic acid (JEOL-JEM- 2100, Japan).

The entrapment efficiency (EE%) and loading efficiency (LE%) were calculated by centrifuging a 1-mL NCs suspension for 30 min at 18,000 rpm at 4 °C (Model 3 K 30, Sigma, Germany) and then measuring the unentrapped extract in the supernatant using a UV–visible spectrophotometer at 285 nm (Shimadzu UV 1650 Spectrophotometer, Japan). The following equations were used to calculate EE% and LE%:

$$\begin{aligned}\mathrm{EE}\%= & \left(\mathrm{weight\ of\ loaded\ ASH\ extract} / \right. \\ & \left.\mathrm{weight\ of \ initial\ ASH\ extract}\right)\times 100\end{aligned}$$

$$\begin{aligned}\mathrm{LE}\%= & \left(\mathrm{weight\ of\ ASH\ extract}/ \right. \\ & \left. \mathrm{weight\ of\ NCs}\right)\times 100\end{aligned}$$

Thermal analysis of NCs and their ingredients was conducted using a differential scanning calorimeter (DSC, Model DTG-60H, Shimadzu, Japan); 5 mg of each sample was placed in sample pans and heated from 30 to 350 °C with a heating rate of 10 °C/min under nitrogen purge using an empty pan as reference [31].

The dissolution profile was measured using the dialysis technique. Briefly, 1 mL of NCs suspension was placed in the donor compartment of a diffusion cell, covered by a cellulose membrane (cutoff 12,000 Mw), and immersed in the receiver compartment, which contained 100 mL of 0.1 N HCl or phosphate buffer saline (PBS, pH 7.4). One percent w/v Tween 80 was added to the dissolution media to maintain sink condition. At various time intervals, 1-mL aliquots were withdrawn and replaced with a fresh medium. Aliquots were analyzed using a UV–visible spectrophotometer at 285 nm. The cumulative dissolution profile and model fitting kinetics were then estimated [35, 36].

### Evaluation of ASH-BPNC in an HE rat model

#### Animals

Thirty-two adult female Wistar rats (7–8 weeks old, 180–210 g) were obtained from the Faculty of Veterinary Medicine, Cairo University (Giza, Egypt). The study design was approved by the Veterinary Institutional Animal Care and Use Committee (approval no:Vet CU20022020157) and agreed with the AVMA Guidelines for the Euthanasia of Animals: 2013 Edition [37]. Rats were kept in the animal unit facility at room temperature 22-25 °C with constant humidity, and a 12:12-h dark:light cycles. They had free access to food and water and were acclimatized for 1 week before study initiation.

#### Experimental design and induction of HE

Rats were randomly allocated into 4 groups of 8 rats each as follows:

- Group 1 (Normal control): Normal rats were given saline (2 mL/kg b.w) via oral gavage.
- Group 2 (TAA): Rats were given saline orally for 14 consecutive days, followed by TAA (350 mg/kg, once, ip) (Sigma Chem., USA), and continued with saline for another 7 successive days [14].
- Group 3 (ASH, 400 mg/kg): Rats were orally administered ASH (400 mg/kg) for 14 consecutive days, followed by TAA (350 mg/kg, once, ip), and continued with ASH (400 mg/kg) for another 7 consecutive days [14].
- Group 4 (ASH-BPNC, 100 mg/kg): Rats were orally administered ASH-BPNC (100 mg/kg) for 14 consecutive days, followed by TAA (350 mg/kg, once, ip), and continued with ASH-BPNC (100 mg/kg) for another 7 consecutive days.

The dose of ASH-BPNC was selected on the basis of our preliminary study. All animals were provided with a 5% dextrose solution and Ringer’s lactate as supportive therapy to avoid hypoglycemia and renal failure. The survival rate and health status of rats were monitored throughout the experiment.

#### Neurological scoring of HE

Forty-eight hours following TAA administration, each rat was subjected to neurological scoring by a professional observer. The scoring system consisted of a series of sensory tests, including withdrawal, auditory startle, head shake, corneal, and righting reflexes; equilibrium test; grasping; and placement, according to Fadillioglu et al. [38] with some modifications. The score in each sensory test ranged from 0 to 3, where 0 indicates no response, 1 indicates mild response, 2 indicates moderate response, and 3 indicates normal response.

#### Assessment of behavioral alterations

Behavioral tasks were conducted from day 22 to 26 of the experiment, between 09:00 a.m. and 3:00 p.m. The rats were adapted to the testing room before the behavioral assessment. In this assessment, motor activity and cognitive functions were evaluated as follows:

##### Motor activity

The motor activity of rats was assessed in an open field apparatus for 3 min, as previously described [39]. The testing parameters were the number of crossing squares and the rearing frequency.

##### Cognitive function

The cognitive functions of the rats, including spatial and nonspatial working memory, were evaluated using a Y-maze and a novel object recognition task, as formerly described [14, 39]. Y-maze response was measured using the spontaneous alternation percentage (SAP), while the novel object recognition task was assessed using the total exploration and novel object preference percentage.

#### Sample collection

Twenty-four hours after the behavioral tests, blood samples were collected from the retro-orbital plexus, left to clot, and centrifuged at 4000 rpm for 10 min for serum separation. Next, rats were sacrificed, and liver and brain samples were excised. Parts of liver and brain tissues were stored at −80 °C for subsequent gene expression and biochemical analyses, and the remaining tissues were fixed in 10% buffered formalin for histopathological and immunohistochemical examinations.

#### Biochemical analyses

The serum levels of aspartate aminotransferase (AST) and alanine aminotransferase (ALT), as well as levels of reduced glutathione (GSH) and malondialdehyde (MDA) in liver and brain tissue homogenates were assayed using commercial kits purchased from Biodiagnostic Co., (Giza, Egypt). In addition, glutamine synthetase (GS), HO-1, and the nuclear levels of Nrf2 were assessed in the supernatants of the homogenized liver and brain tissue samples using the commercially available enzyme-linked immunosorbent assay (ELISA) kits (Northwest Life Science Specialties, LLC, WA, USA). The protein contents in tissues were determined using the Bradford protein assay kit (Genei, Bangalore).

#### Quantitative reverse transcription-polymerase chain reaction (PCR) analyses

Liver and brain tissue samples (100 mg) were homogenized in 1 mL of lysis buffer. Total RNA was extracted, from the homogenized tissues, using easy-spin^™^ (DNA free) Total RNA Extraction Kit (Intron Biotechnology, Korea). Its purity and concentration were assessed using a NanoDrop (Thermo Fischer Scientific, USA). Reverse transcription was then carried out using a RevertAid First Strand cDNA Synthesis Kit (Thermo Scientific, USA).

The expression levels of Nrf2, NQO1, GCLC, ERK1/2, and p38 genes were analyzed by StepOne^™^ Real-Time PCR (Applied Biosystems, USA) using Maxima SYBR Green qPCR Master Mix, no ROX (2X) (Thermo Scientific, USA). The primer sequences were Nrf2 (F:5′- CACATCCAGACAGACACCAGT-3′, R:5′- CTACAAATGGGAATGTCTCTGC); NQO1 (F:5′-GCCATGAAGGAGGCTGCTGT-3′, R:5′-ATCACCAGGTCTGCAGCTTC-3′); GCLC (F:5′-GTGGACACCCGATGCAGTAT-3′, R:5′-TCATCCACCTGGCAACAGTC-3′); ERK1/2 (F:5′-TCAAGCCTTCCAACCTC-3′, R:5′-GCAGCCCACAGACCAAA-3′); p38 (F:5′-ACATCGTGTGGCAGTGAAGAAG-3′, R:5′-CTTTTGGCGTGAATGATGGA-3′); GAPDH (F:5′-AGGTTGTCTCCTGTGACTTC-3′, R:5′-CTGTTGCTGTAGCCATATTC-3′). Negative controls were also prepared. PCR was then performed with initiation at 95 °C for 5 min, followed by 40 cycles of denaturations at 95 °C for 30 s, annealing at 58 °C for 30 s, and extension at 72 °C for 30 s using. GAPDH was used as a reference gene.

#### Histopathological and immunohistochemical examinations

Liver and brain tissues were fixed in 10% neutral formalin saline and embedded in paraffin wax. Tissue sections (5 µm) were cut and stained with hematoxylin and eosin (H&E) for light microscopy [40]. The liver was histologically scored as previously described by Bruck et al. [41].

Masson’s trichrome stain (MTC) was used to evaluate the extent of fibroplasia in liver tissue and was quantified as area percentage using cellSens dimensions software (Olympus), as previously described [42].

Formalin-fixed paraffin-embedded liver and brain blocks were used for immune staining, as previously described [14]. Briefly, Sects. (5 µm) were subjected to rehydration, heat-induced epitope retrieval, protein blocking, and endogenous peroxidase blocking followed by incubation with primary antibodies overnight at 4 °C:mouse monoclonal anti-nuclear factor (NF)-κB, anti-tumor necrosis factor (TNF)-α, and anti-glial fibrillary acidic protein (GFAP, Santa Cruz Biotechnology, Inc.). After washing with phosphate buffer saline, an HRP-labeled secondary antibody (Abcam, UK) was applied for 2 h at room temperature. The reaction was visualized using the DAB-substrate detection kit. Secondary-only control slides were generated by leaving out the primary antibody step. Slides were imaged using an Olympus DP-27 camera fitted to an Olympus BX43 microscope, and positive expression was quantified as area percentage.

####  In vivo biodistribution studies

The fluorescent marker, fluorescein diacetate dye (FDA) (Sigma Chem., USA), was incorporated into ASH-BPNC. Then, ASH-BPNC loaded with FDA (ASH-BPNC-FDA) was orally administered (equivalent to 100 mg/ kg of ASH) to normal rats (*n* = 6). After 12 h, animals were sacrificed, and 100 mg of vital organs (brain, liver, kidney, and lungs) were excised and homogenized in ice-cold PBS. The accumulation of ASH-BPNC-FDA was detected in the resulting tissue homogenates using spectrofluorometer (PerkinElmer, Germany) at ʎexc:490 nm and ʎem:520 nm, respectively.

Furthermore, the accumulation of ASH-BPNC-FDA in tissues of vital organs were confirmed using in vivo fluorescent imaging, where sections of the remaining parts of the excised vital organs (brain, liver, kidney, and lungs) were preceded for Tissue-Tek Cryosat Microtome (Thermo Fisher, USA). Sections were examined under fluorescent microscope (IX81, Olympus, Tokyo, Japan) at magnification power × 200, and photomicrographs were captured.

#### Statistical analyses

Results were represented as mean (*n* = 8) ± standard deviation (SD). Parametric data were statistically analyzed using one-way analysis of variance (ANOVA) followed by the Tukey multiple comparison test as a post hoc test, whereas the Kruskal–Wallis test followed by Dunn’s post hoc was used for nonparametric analysis, which was represented as median (minimum and maximum). Moreover, the survival rate was analyzed using the Kaplan–Meier test. Statistical analyses were performed using GraphPad Prism, version 6. Test for normality was carried out for our data.

## Results

### Preparation and characterization of ASH-BPNC

#### Preparation of ASH-BPNC

**Fig. 1:**
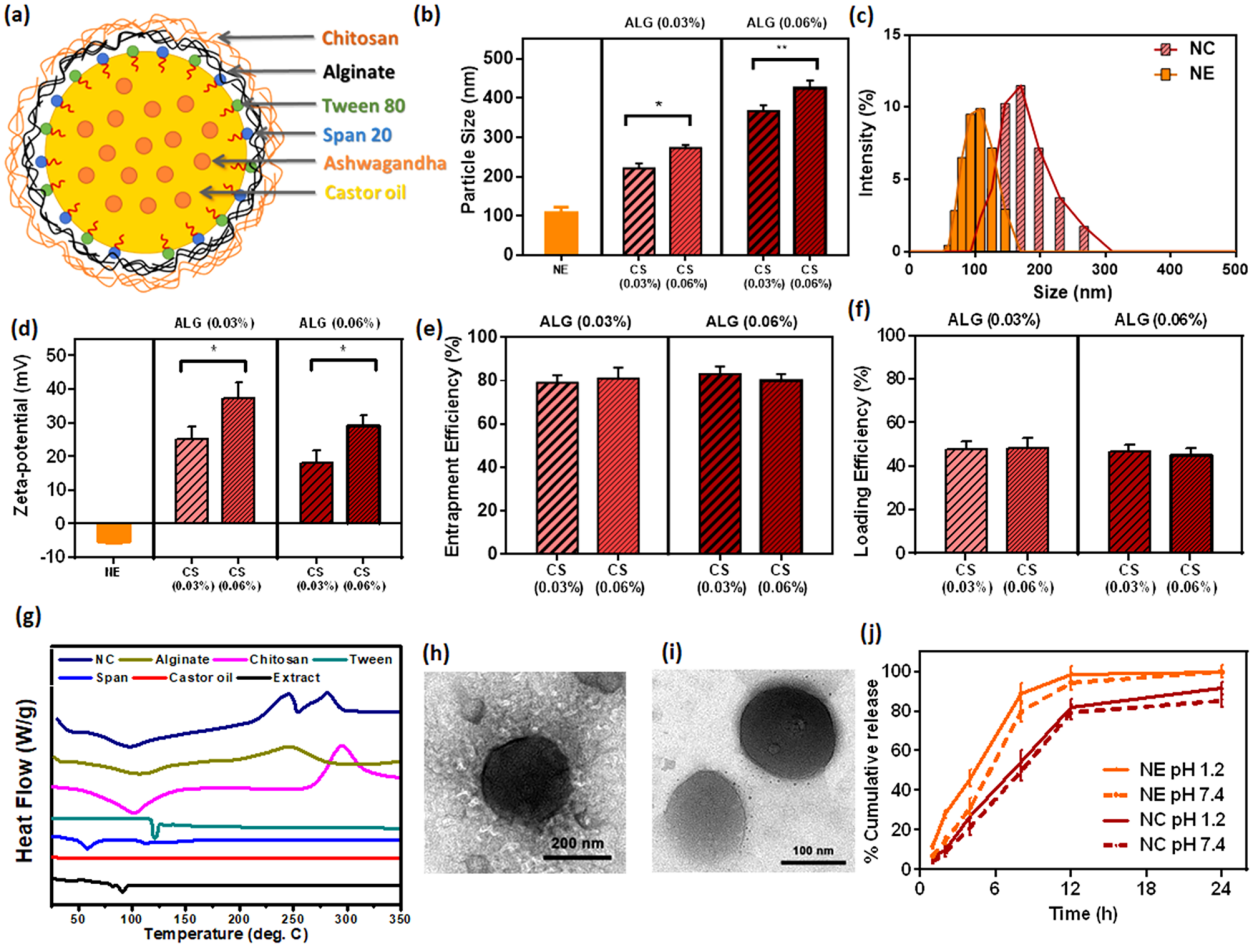
Development of ASH-BPNC: A schematic illustration of NC structure with a NE core and bipolymeric shell (**a**), particle size of the developed NE and NC (**b**), histogram of the particle size distribution of NE and NC (**c**), zeta potential of NE and NC (**d**), entrapment efficiency (**e**), loading efficiency (**f**), DSC thermograms (**g**), representative TEM image of the selected NC (**h**) and NE (**i**), and drug dissolution profile of the selected NE and NC in neutral and acidic medium (**j**). Data are represented as mean ± SD (*P* < 0.05 and *P* < 0.01). ALG and CS refer to sodium alginate and chitosan, respectively

ASH-BPNCs were prepared using emulsification followed by ionic gelation process. The NCs consisted of NE core and a bipolymeric shell, as illustrated in Fig. 1a.

#### Physical characteristics of NCs

The physical properties of different NC formulations were investigated using the dynamic light scattering technique. The particle size of uncoated NE was 108 ± 13 nm. Four formulations were prepared, where alginate and chitosan were used at two concentrations (0.03% or 0.06% w/v). The resultant particle size is demonstrated in Fig. 1b. Increasing chitosan from 0.03 to 0.06% resulted in a significant increase in particle size from 221 ± 13 to 273 ± 8 nm for 0.03% alginate (*P* < 0.05) and from 367.1 ± 15 to 425.2 ± 20 nm for 0.06% alginate (*P* < 0.01), with a polydispersity index ranging from 0.22 to 0.43. The size distribution histogram of NE and NC formulas of 0.03% alginate and 0.03% chitosan showed an increase in particle size after coating, indicating successful coating of polymer (Fig. 1c). These results are in agreement with Natrajan et al. [32].

The zeta-potential for uncoated NE and NC was −5.2 mV and > 20 mV for all formulations, respectively, indicating a stable colloid system due to the charge repulsion effect. A higher zeta-potential was observed at low alginate (negatively charged) concentration (Fig. 1d**)**. Increasing the chitosan concentration increased the charge from 25.2 to 37.2 mV at low concentration of alginate and from 18.1 to 29.2 mV at high concentrations of alginate. The EE% for all formulations was approximately 80%, as shown in Fig. 1e. The LE% for all formulations was approximately 45%, as shown in Fig. 1f. The NC formula of 0.03% alginate and 0.03% chitosan was selected, where particle size, zeta-potential, and EE% were 221 ± 13 nm, 25.2 mV, and 79%, respectively.

Differential scanning calorimetry (DSC) is a standard technique used to detect the thermal properties of nanosystems. Figure 1g shows an endothermic peak at 92.5 °C for the extract, no significant peak for castor oil, 59.5 °C for Span 20, 116.2 °C for Tween 80, and a broad endothermic peak around 106 °C, which could be owed to unbound water loss. There was an exothermic peak at 251.8 °C for alginate, a broad endothermic peak approximately 100 °C resulting from unbound water loss, and an exothermic peak of 295 °C for chitosan. Thermograms of NCs exhibited one elongated endothermic peak similar to the characteristic alginate and chitosan peaks and two exothermic peaks at 243 °C and 282 °C. These results confirmed the assembly of NCs, with detection of both chitosan and alginate peaks.

#### Morphological characteristics using TEM

TEM showed the NCs as spherical particles with a diameter of approximately 250 nm and a thin surrounding shell (Fig. 1h). Also, the morphological characteristics of uncoated nanoemulsion were showed spherical particles (Fig. 1i).

#### Dissolution and in vitro release profile

The dissolution profile of the selected formula was investigated at acidic and neutral pH while maintaining sink condition using 1% Tween 80, as shown in Fig. 1j. Using solutions of different pH showed no effect on the dissolution profile of both the NEs and NCs. The in vitro release profile of NE revealed that almost 40% of the extract was released after 4 h and reached more than 90% after 12 h. On the other hand, the in vitro release profile of NC revealed that almost 25% of the extract was released after 4 h and reached more than 80% after 12 h, and approximately 90% of the extract was released after 24 h. These data confirmed the ability of the bipolymeric shell to control release of the extract. There was a slight decrease in the dissolution profile at pH 7.4, which could be attributed to an insoluble layer of chitosan forming at neutral pH, because chitosan is soluble in acidic medium. The dissolution profiles were fitted to several kinetic models, and the Hixson–Crowell model was found to be the most suitable (*r*2 = 0.96) [43].

### Assessment of ASH-BPNC hepatoprotective and neuroprotective activity in the HE model

#### Effects of ASH-BPNC on the survival and neurological scores of HE

**Fig. 2:**
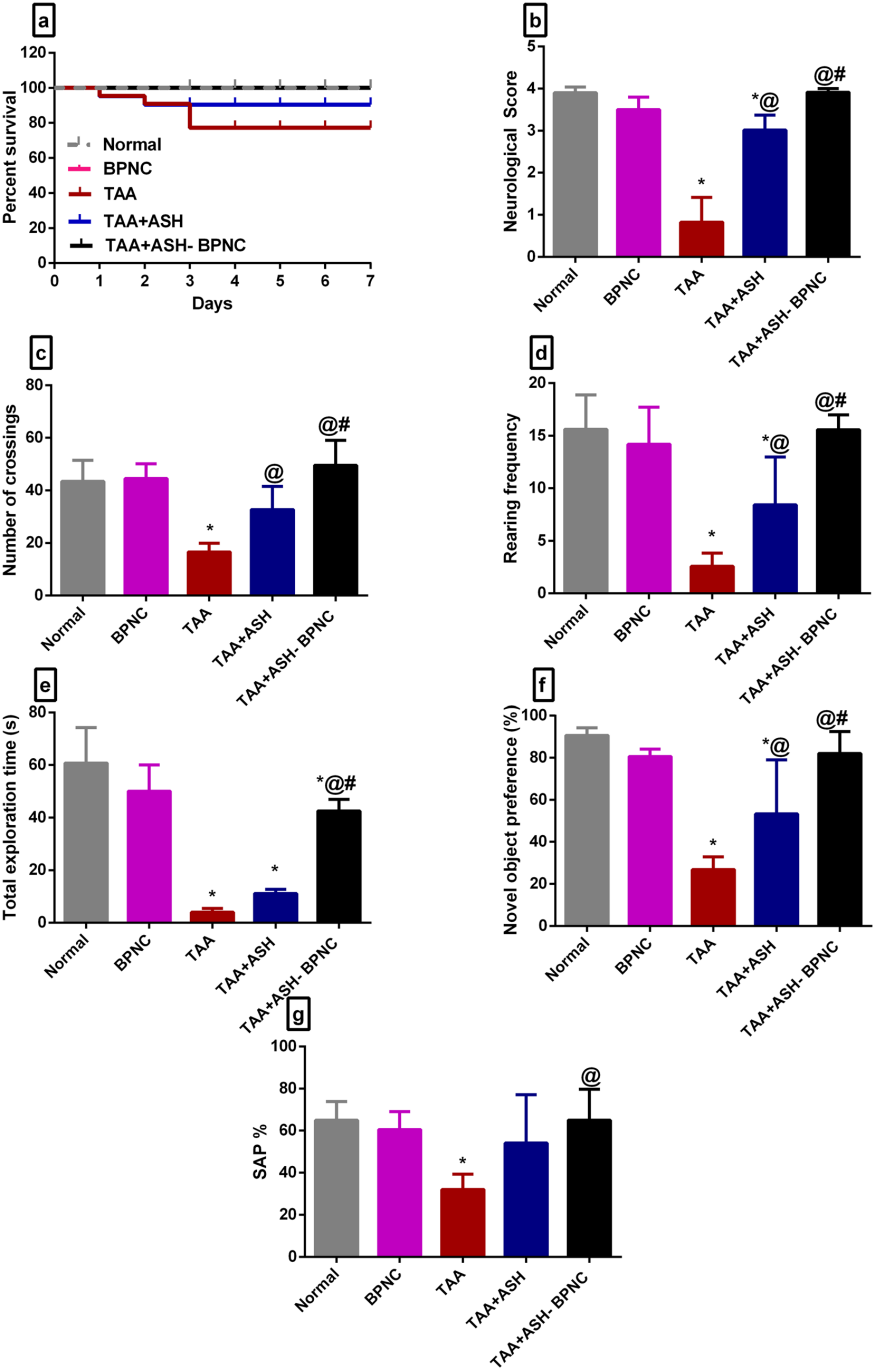
Effects of ASH-BPNC on survival, neurological scores, motor activity, and cognitive task performance in TAA-induced HE in rats. Percent survival (**a**), neurological score (**b**), number of crossings squares in open field (**c**), rearing frequency in open field (**d**), total exploration time in novel object recognition task (**e**), novel object preference percentage (**f**), and spontaneous alternation percentage in Y-maze (**g**). Data are represented as mean ± SD (*P* < 0.05),*significant from normal control; ^@^significant from TAA-intoxicated group; ^#^significant from TAA + ASH-treated group

TAA-intoxicated rats displayed a marked low survival and neurological score when compared with normal rats. Treatment with ASH showed an enhanced survival rates and neurological scores when compared with TAA-intoxicated rats. Interestingly, ASH-BPNC-treated rats showed a survival rate and neurological score superior to ASH-treated rats (Fig. 2a, b). Treatment with BPNC alone displayed no significant effects on either survival or neurological scores of HE when compared with normal rats.

#### Effects of ASH-BPNC on the behavioral alterations caused by TAA intoxication

TAA intoxication resulted in a significant reduction in the general motor activity in the open field task, as visualized by the decline in the number of crossing squares and rearing frequencies compared with normal rats. The administration of either ASH or ASH-BPNC significantly increased the general motor activity with superior results in the ASH-BPNC-treated group (Fig. 2c, d).

Concerning cognitive tasks, TAA-intoxicated rats showed significant cognitive deficits as visualized by a marked reduction in the total exploration time and novel object preference percentage in the novel object recognition tasks. Additionally, TAA intoxication decreased SAP in the Y-maze task compared with normal control group. ASH treatment resulted in a significant increase in SAP in the Y-maze task when compared with TAA-intoxicated rats. The administration of ASH-BPNC restored the cognitive abilities (Fig. 2e–g). Treatment with BPNC alone displayed no effects on cognitive abilities when compared with normal rats.

#### Effects of ASH-BPNC on hepatotoxicity markers and serum ammonia levels

**Fig. 3:**
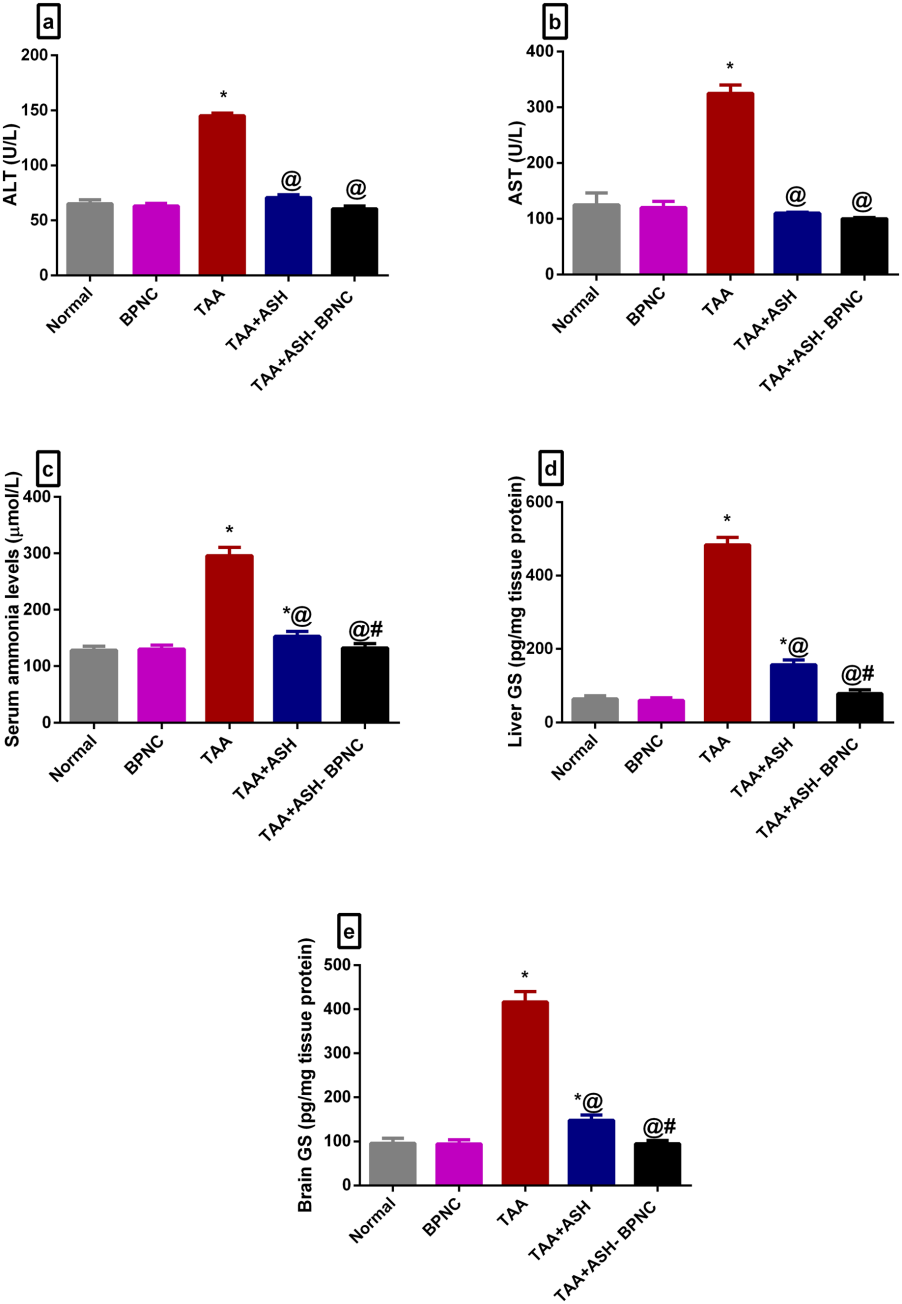
Effects of ASH-BPNC on hepatotoxicity markers, serum ammonia levels, and glutamine synthetase (GS) in TAA-induced HE in rats. Serum levels of ALT (**a**), AST (**b**), ammonia (**c**), hepatic (**d**), and brain (**e**) levels of GS. Data are represented as mean ± SD (*P* < 0.05). *significant from normal control; ^@^significant from TAA-intoxicated group; ^#^significant from TAA + ASH-treated group

TAA-induced ALF was manifested by a marked increase in ALT and AST serum levels when compared with normal rats (Fig. 3a, b). ASH administration significantly lowered the elevated ALT and AST levels compared with the TAA-intoxicated group. However, ASH-BPNC restored the levels to normal.

TAA intoxication induced a pronounced elevation in the serum levels of ammonia (Fig. 3c). This was accompanied by a marked elevation in hepatic and brain levels of GS compared with normal rats (Fig. 3d, e). ASH significantly reduced the elevated GS levels compared with TAA-intoxicated rats, whereas the administration of ASH-BPNC (100 mg/kg) restored the levels to normal. Administration of BPNC alone displayed no effects on either liver functions or ammonia levels compared with normal control group.

#### Effects of ASH-BPNC on the TAA-induced histopathological alterations in liver and brain tissues

**Fig. 4:**
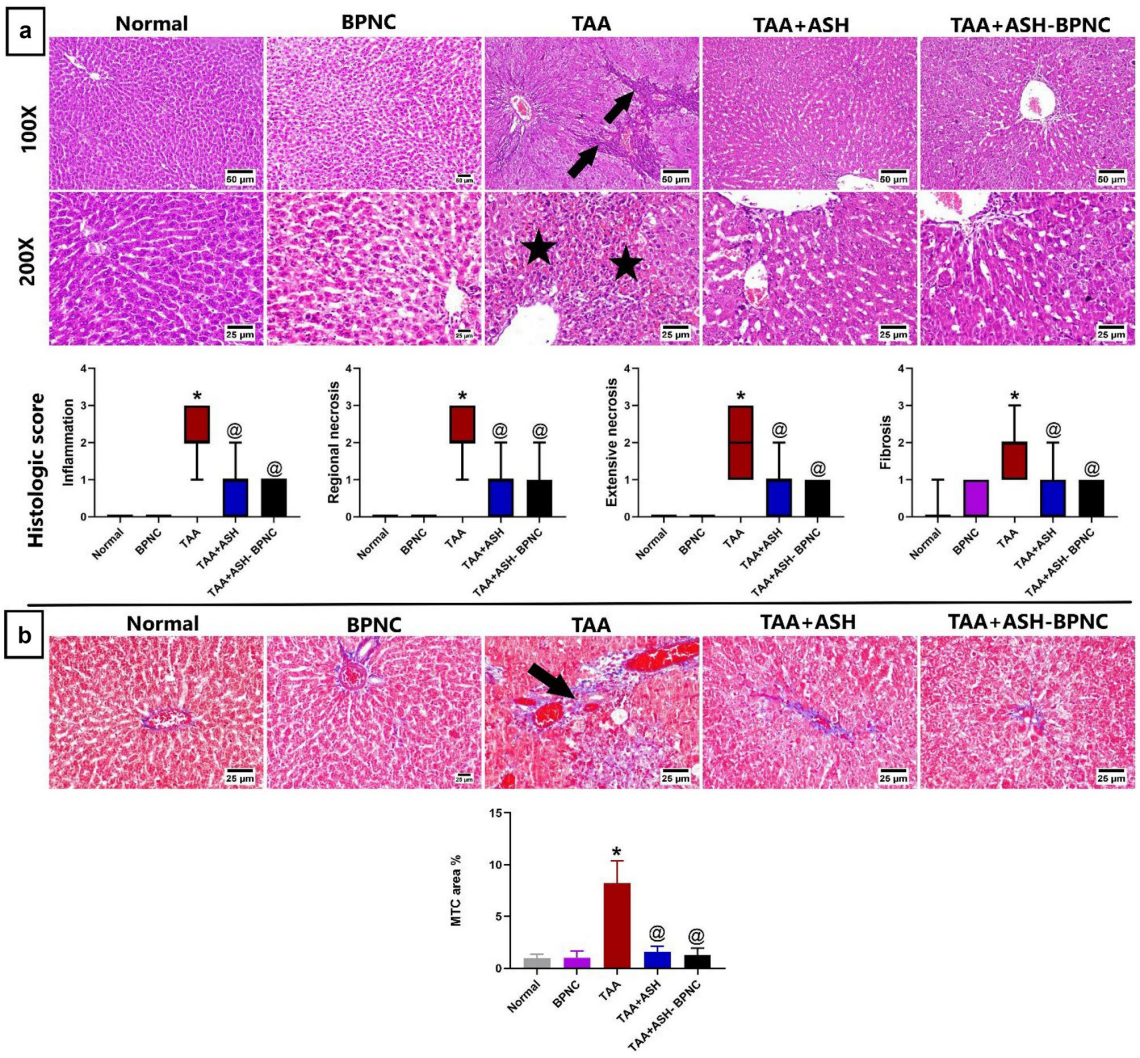
Effects of ASH-BPNC on hepatic histopathological alterations in TAA-induced HE in rats. Photomicrographs of hepatic sections stained with H&E (**a**) showing normal histology of hepatic parenchyma in normal and BPNC groups; TAA-intoxicated hepatic sections showing portal fibroplasia with mononuclear inflammatory cells infiltration (arrows) and extensive hepatocellular necrosis and hemorrhage; TAA-intoxicated hepatic sections treated with ASH showing apparently normal liver cells with few mononuclear inflammatory cells infiltration; TAA-intoxicated hepatic sections treated with ASH-BPNC showing apparently normal hepatocytes (upper panel); and a chart of liver histologic score (lower panel) with data represented as median (Max. and Min.) (*P* < 0.05). Photomicrographs of hepatic sections stained with MTC (**b**) showing TAA-intoxicated hepatic sections with mild portal fibroplasia in group (arrow) and TAA-intoxicated hepatic sections treated with either ASH or ASH-BPNC showing apparently normal liver histology (upper panel) and a chart showing MTC-stained area (lower panel) represented as mean ± SD (*P* < 0.05). *significant from normal control group; ^@^significant from TAA-intoxicated group; ^#^significant from TAA + ASH-treated group

Liver tissues of the TAA-intoxicated group exhibited portal fibroplasia with heavy infiltration of mononuclear inflammatory cells and extensive hepatocellular necrosis with marked parenchymal loss. In rats administered ASH, there were only mild inflammatory reactions in few hepatic sections, whereas the administration of ASH-BPNC showed apparently normal hepatocytes. The estimated histologic scores of the hepatic sections were significantly higher in the TAA intoxicated group than in normal group. Both ASH- and ASH-BPNC-treated groups had considerable reduction in the histological damage and fibroplasia of liver, although this was not statistically significant (Fig. 4).

**Fig. 5:**
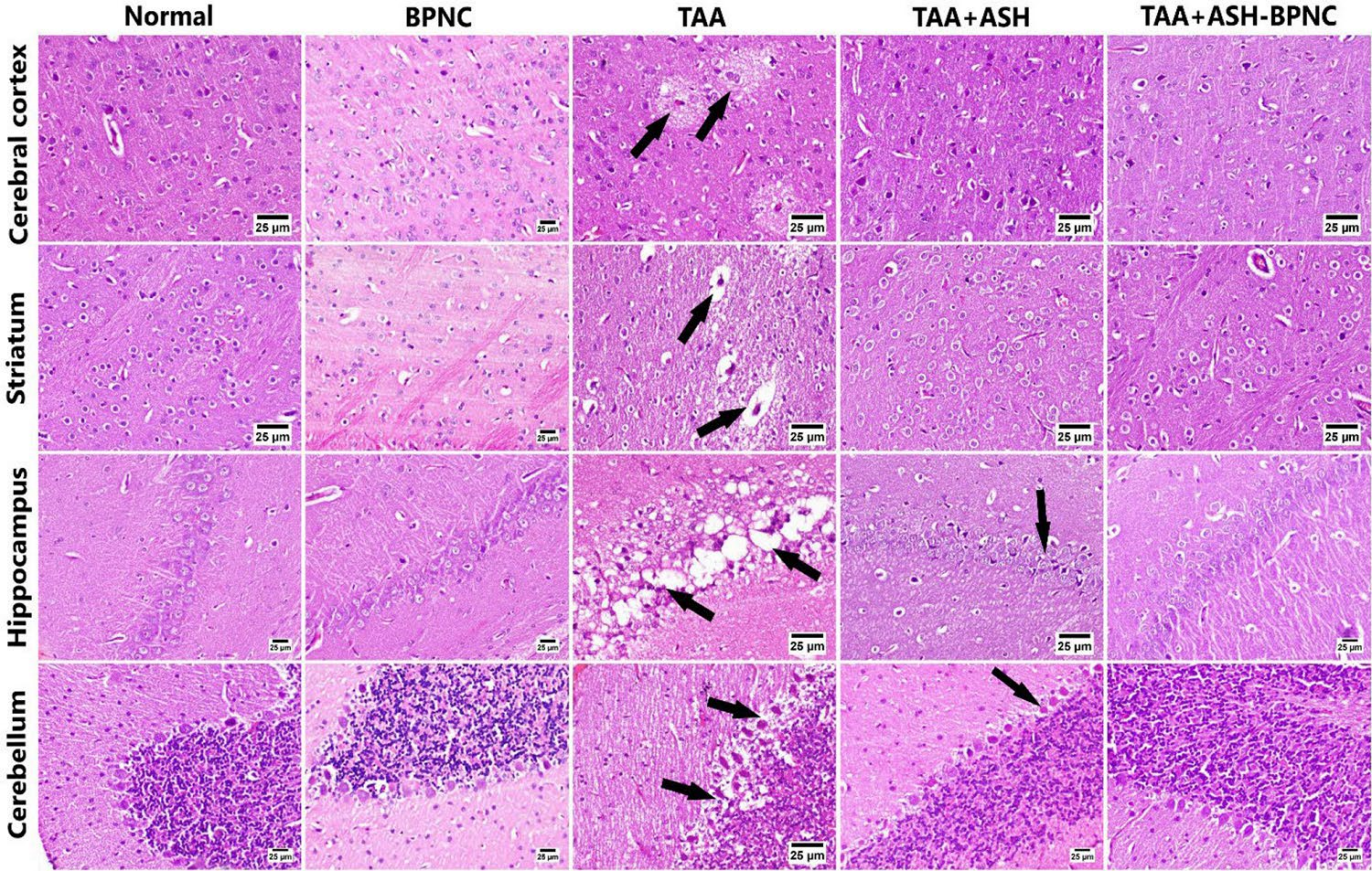
Effects of ASH-BPNC on brain histopathological alterations in TAA-induced HE in rats. Photomicrographs of brain sections stained with H&E displaying TAA-intoxicated brain sections with area of vacuolation and neuronal degeneration in cerebral cortex (arrows), edema and demyelination in striatum (arrows), extensive vacuolation and neuronal necrosis in hippocampus (arrows), and Purkinje cell necrosis with vacuolation in the cerebellum (arrows); TAA-intoxicated brain sections treated with ASH showing few dark neurons in the cerebral cortex, apparently normal striatum and few degenerating cells in hippocampus (arrows), and cerebellum (arrows); TAA-intoxicated brain sections treated with ASH-BPNC showing apparently normal cerebral cortex, striatum, hippocampus, and cerebellum

In addition, marked histopathological alterations were detected in brain tissues of the TAA-intoxicated group (Fig. 5). The cerebral cortex showed neuronal necrosis with obvious vacuolation, astrocytosis, and edema. Similarly, the striatum exhibited neuronal edema with demyelination. Neurons within the hippocampus, especially those of the CA-1 region, showed signs of degeneration, necrosis, and marked vacuolation. Vacuolation was also apparent in the cerebellum, as well as Purkinje cell necrosis. Treatment of the TAA-intoxicated group with ASH caused remarkable improvement in the deteriorated brain tissues, with only a few dark degenerated neurons visible within the cerebral cortex, hippocampus, and cerebellum and with apparently normal striatum. Notably, the ASH-BPNC-treated group displayed normal histopathology in all brain regions, indicating better neuroprotective effects than ASH. Administration of BPNC alone manifested no histopathological effects on hepatic and brain tissues when compared with normal control group.

#### Effects of ASH-BPNC on the oxidative stress markers and the Nrf2 pathway in hepatic and brain tissues

**Fig. 6:**
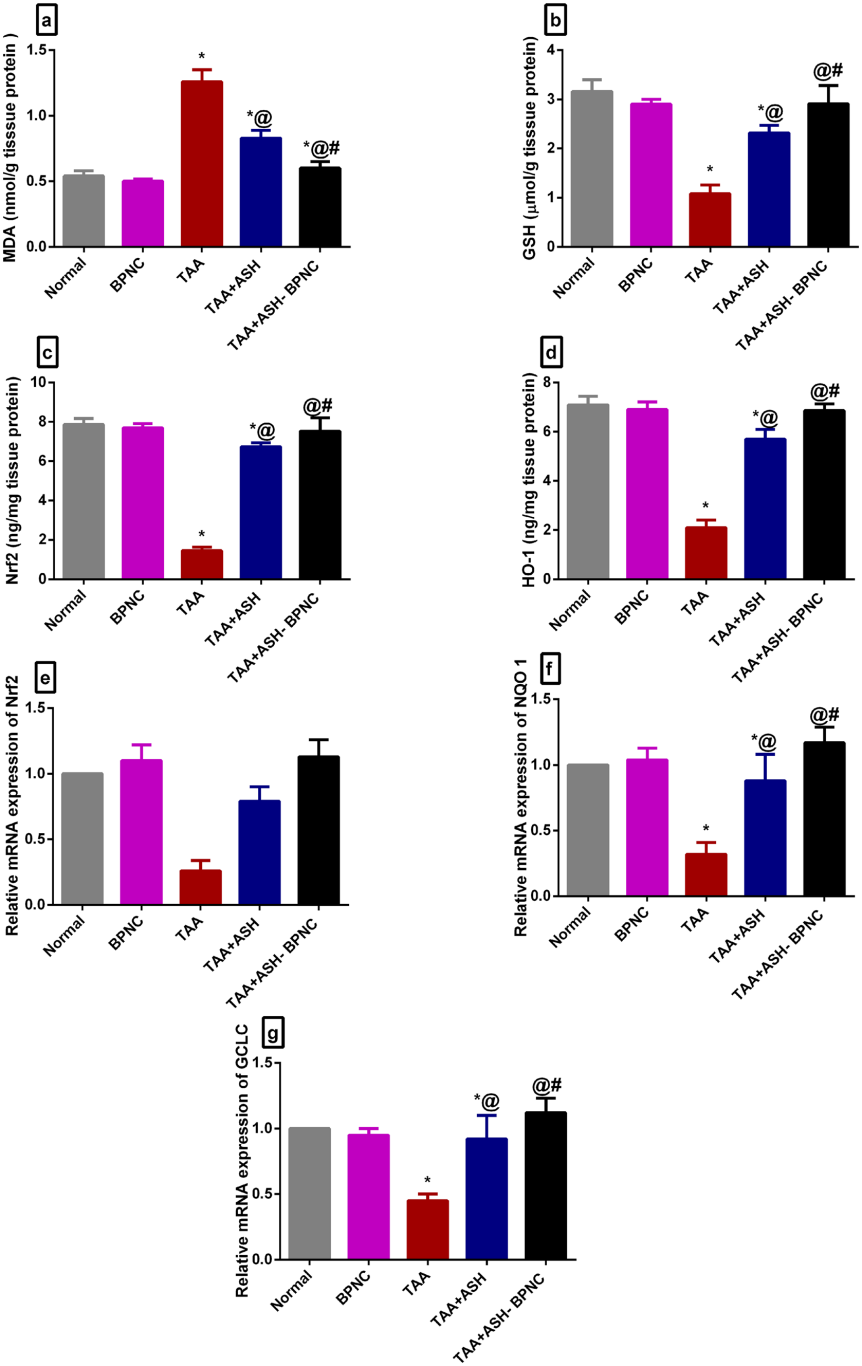
Effect of ASH-BPNC on hepatic oxidative stress markers and Nrf2 pathway in TAA-induced HE in rats. Hepatic tissue levels of MDA (**a**), GSH (**b**), Nrf2 (**c**), and HO-1 (**d**); relative mRNA expression levels of Nrf2 (**e**), NQO1 (**f**), and GCLC (**g**) in hepatic tissues. Data are represented as mean ± SD (*P* < 0.05). *significant from normal control, ^@^significant from TAA-intoxicated group, and ^#^significant from TAA + ASH-treated group

**Fig. 7:**
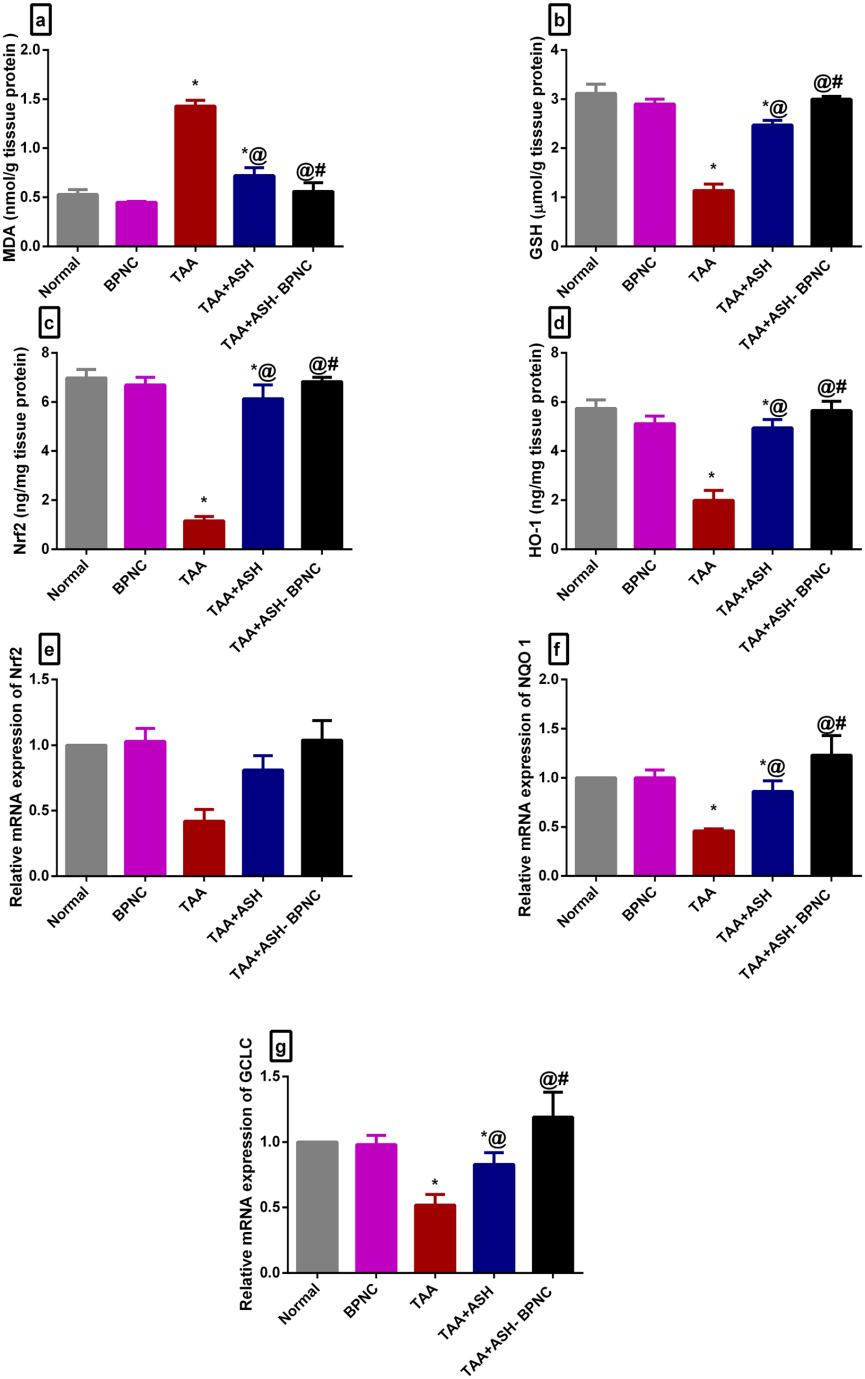
Effect of ASH-BPNC on brain oxidative stress markers and Nrf2 pathway in TAA-induced HE in rats. Brain tissue levels of MDA (**a**), GSH (**b**), Nrf2 (**c**), and HO-1 (**d**); relative mRNA expression levels of Nrf2 (**e**), NQO1 (**f**), and GCLC (**g**) in brain tissues. Data are represented as mean ± SD (*P* < 0.05). *significant from normal control and BPNC groups, ^@^significant from TAA-intoxicated group, and ^#^significant from TAA + ASH-treated group

TAA intoxication markedly elevated MDA and depleted GSH levels in the liver and brain tissues compared with the normal control group. ASH markedly reduced the elevated MDA levels and increased the GSH levels in both liver and brain tissues. Interestingly, administration of ASH-BPNC restored MDA and GSH levels to normal (Figs. 6a, b and 7a, b).

Next, the effect of ASH-BPNC on the Nrf2 pathway was determined. Compared with normal rats, TAA intoxication caused a significant reduction in the liver and brain levels of Nrf2 and HO-1. Treatment with ASH significantly elevated their liver and brain levels, whereas treatment with ASH-BPNC restored their levels to normal. The ASH-BPNC’s inducing effect on Nrf2 signaling was further confirmed using gene expression analyses. Results showed that treatment with ASH-BPNC counteracted the suppressive effects of TAA on the mRNA expression levels on Nrf2 as well as its target genes NQO1 and GCLC in hepatic and brain tissues (Figs. 6c–g and 7c–g). Administration of BPNC alone showed no effects on the oxidative stress markers and Nrf2 pathway as compared with normal control group in hepatic and brain tissues.

#### Effects of ASH-BPNC on the pro-inflammatory markers' levels in hepatic tissues

**Fig. 8:**
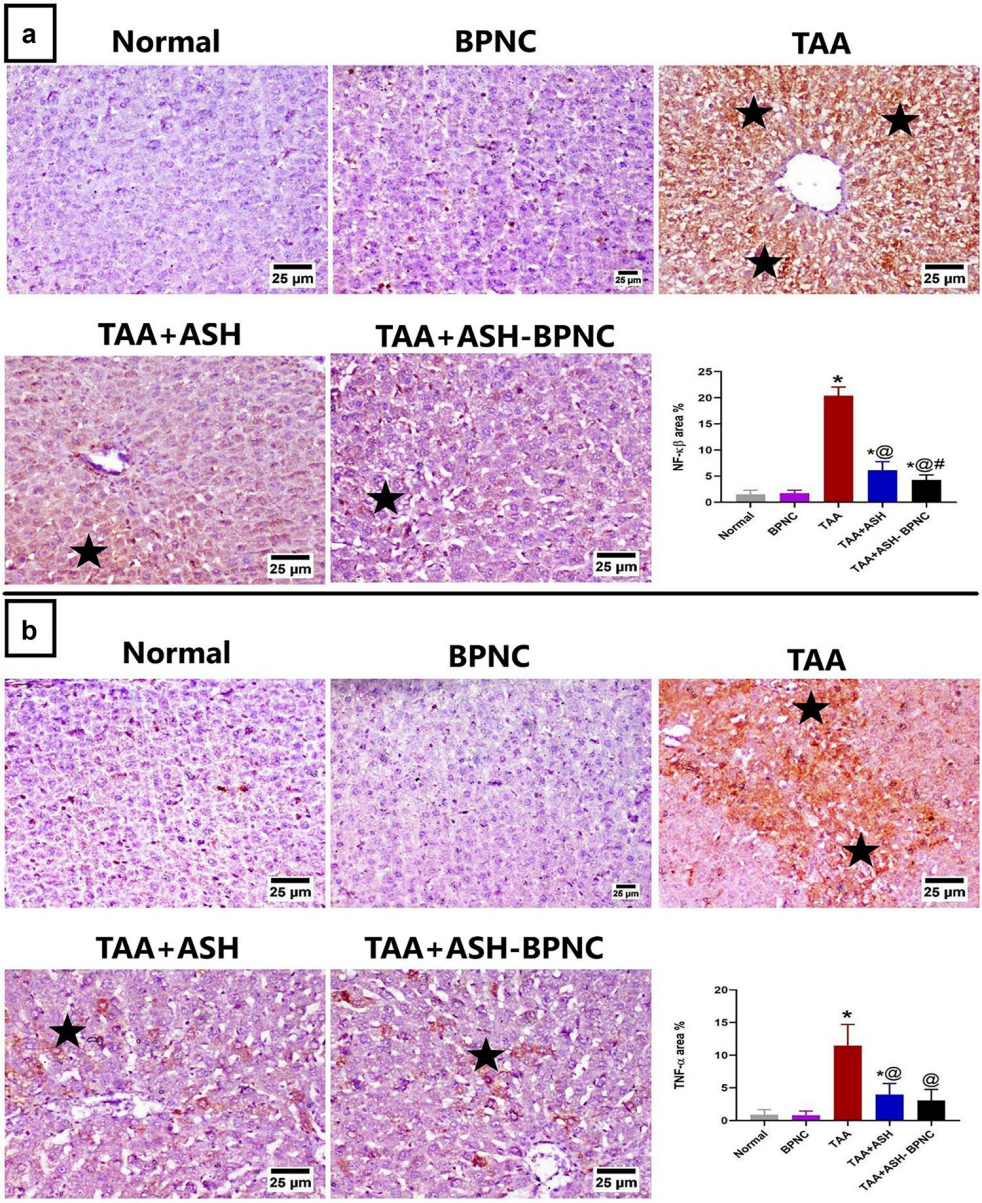
Effect of ASH-BPNC on the hepatic immunohistochemical expression of pro-inflammatory markers (NF-κB and TNF-α) in TAA-induced HE in rats. Representative photomicrographs of NF-κB staining (black stars; positive expression) in hepatic tissues and quantitative analysis of the immunohistochemical expression of NF-κB (left panel). Representative photomicrographs of TNF-α staining (black stars; positive expression) in hepatic tissues and quantitative analysis of the immunohistochemical expression of TNF-α (right panel). Data are represented as mean ± SD (*P* < 0.05). *significant from normal control, ^@^significant from TAA-intoxicated group, and ^#^significant from TAA + ASH-treated group

TAA intoxication induced a pronounced increase in the hepatic immunohistochemical expression of NF-κB and TNF-α. The ASH- and ASH-BPNC-treated groups showed significant downregulation in the immunohistochemical expression of these markers when compared with TAA-intoxicated group. Furthermore, the ASH-BPNC-treated group showed a significant reduction in NF-κB expression when compared with the ASH-treated group. There was no significant difference in hepatic TNF-α expression between the ASH- and ASH-BPNC-treated groups (Fig. 8). Additionally, administration of BPNC alone displayed no effects on the levels of pro-inflammatory cytokines in hepatic tissues when compared with normal control group.

#### Effects of ASH-BPNC on the pro-inflammatory markers and GFAP levels in brain tissues

**Fig. 9:**
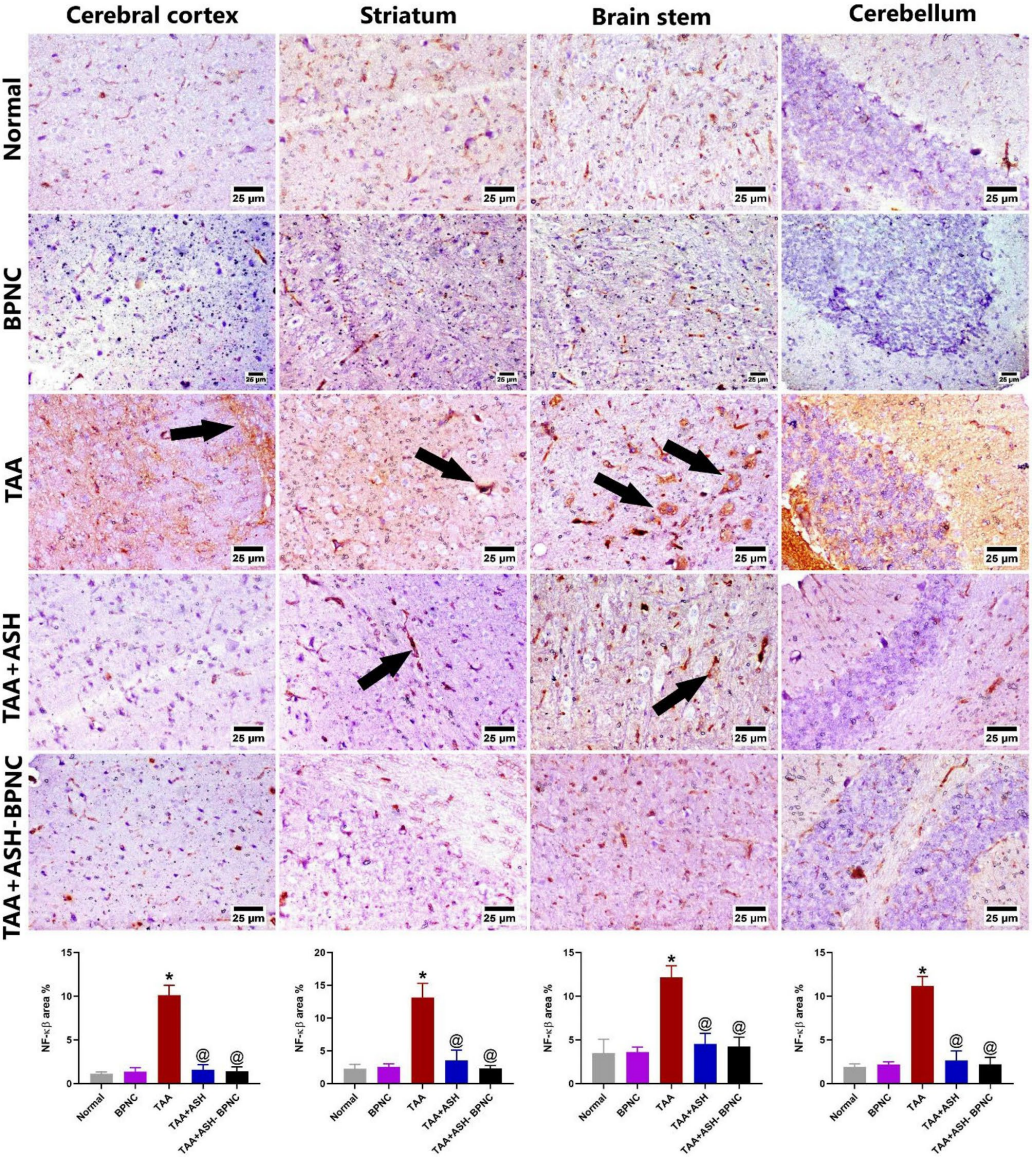
Effect of ASH-BPNC on the brain immunohistochemical expression of NF-κB in TAA-induced HE in rats. Representative photomicrographs of NF-κB staining (black arrows; positive expression) in different regions of brain tissues (upper panel) and quantitative analysis of the Immunohistochemical expression of NF-κB (lower panel). Data are represented as mean ± SD (*P* < 0.05). *significant from normal control; ^@^significant from TAA-intoxicated group; ^#^significant from TAA + ASH-treated group

**Fig. 10:**
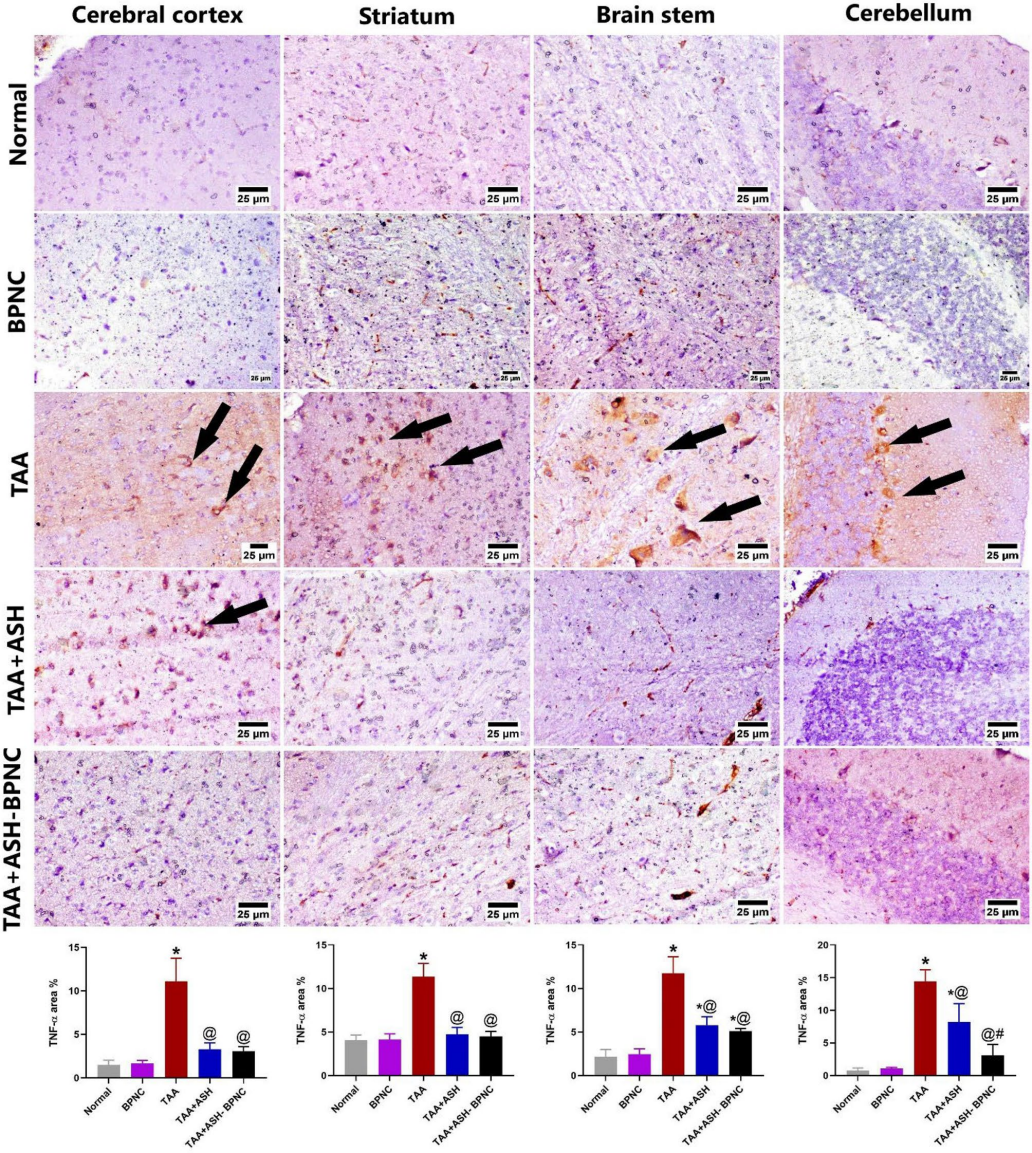
Effect of ASH-BPNC on the brain immunohistochemical expression of TNF-α in TAA-induced HE in rats. Representative photomicrographs of TNF-α staining (black arrows; positive expression) in different regions of brain tissues (upper panel) and quantitative analysis of the immunohistochemical expression of TNF-α (lower panel). Data are represented as mean ± SD (*P* < 0.05). *significant from normal control; ^@^significant from TAA-intoxicated group; ^#^significant from TAA + ASH-treated group

**Fig. 11:**
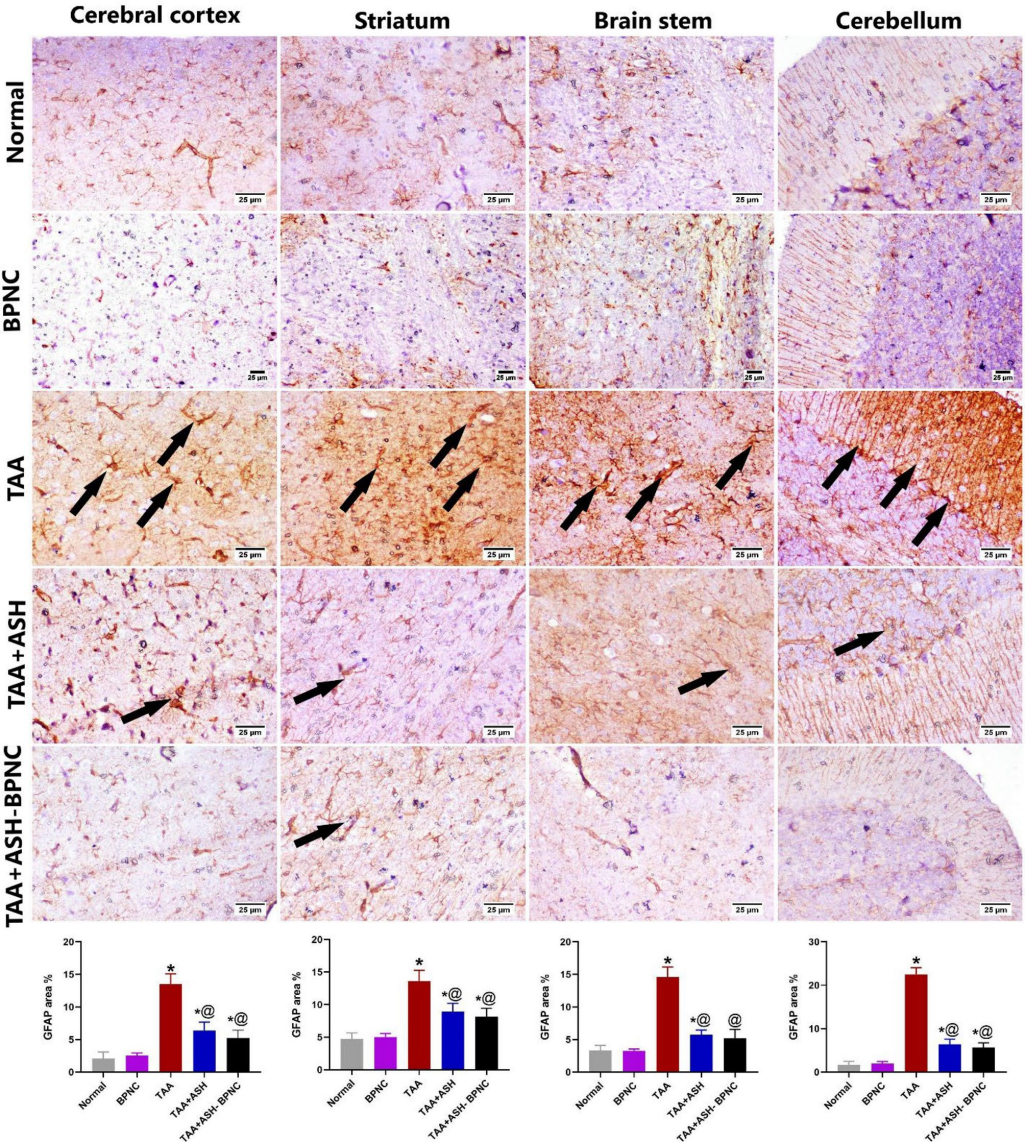
Effect of ASH-BPNC on the brain immunohistochemical expression of GFAP in TAA-induced HE in rats. Representative photomicrographs of GFAP staining (black arrows; positive expression) in different regions of brain tissues (upper panel) and quantitative analysis of the immunohistochemical expression of GFAP (lower panel). Data are represented as mean ± SD (*P* < 0.05). *significant from normal control; ^@^significant from TAA-intoxicated group; ^#^significant from TAA + ASH-treated group

TAA-intoxicated group displayed marked upregulation in the immunohistochemical expression of brain NF-κB, TNF-α, and GFAP compared with the normal control group. Both ASH and ASH-BPNC treatments exhibited significant downregulation in their expressed levels (Figs. 9, 10, and 11). Administration of BPNC alone displayed no effects on the pro-inflammatory cytokines' levels and GFAP expression in brain tissues when compared with normal control group.

#### Effects of ASH-BPNC on the MAPK signaling pathway in hepatic and brain tissues

**Fig. 12:**
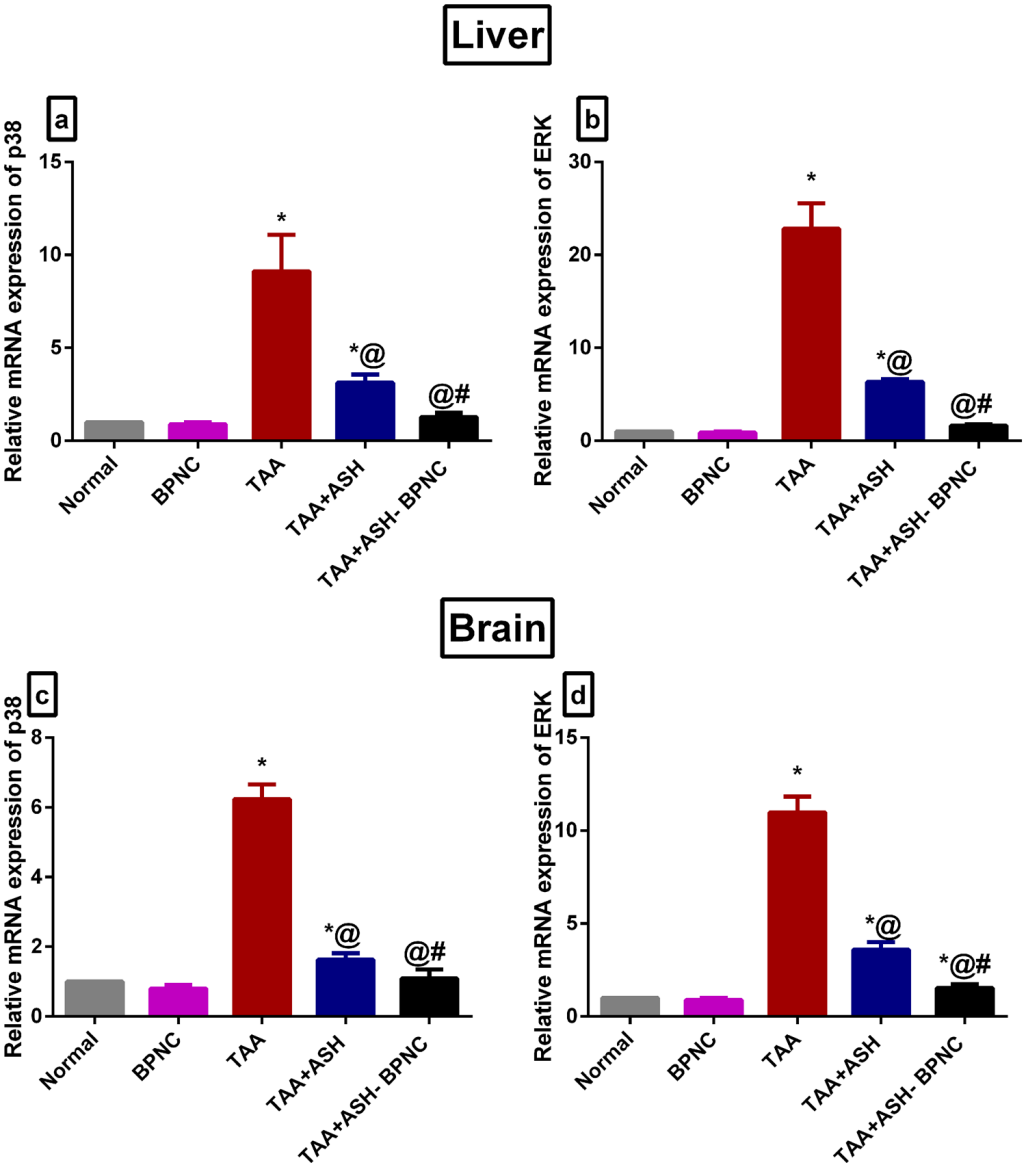
Effect of ASH-BPNC on MAPK signaling pathway in TAA-induced HE in rats. Relative mRNA expression of p38 in hepatic (**a**) and brain (**c**) tissues; Relative mRNA expression of ERK1/2 in hepatic (**b**) and brain (**d**) tissues. Data are represented as mean ± SD (*P* < 0.05). *significant from normal control; ^@^significant from TAA-intoxicated group; ^#^significant from TAA + ASH-treated group

TAA intoxication resulted in the upregulation of mRNA expression levels of p38 and ERK1/2 in both liver and brain tissues when compared with normal rats. The administration of ASH resulted in a significant downregulation in their mRNA expression levels in both liver and brain tissues compared with the TAA-intoxicated group, whereas ASH-BPNC restored the gene expression levels to normal (Fig. 12). Administration of BPNC alone displayed no effects on MAPK signaling in hepatic and brain tissues when compared with normal control group.

#### Effects of ASH-BPNC on organs’ biodistribution

**Fig. 13:**
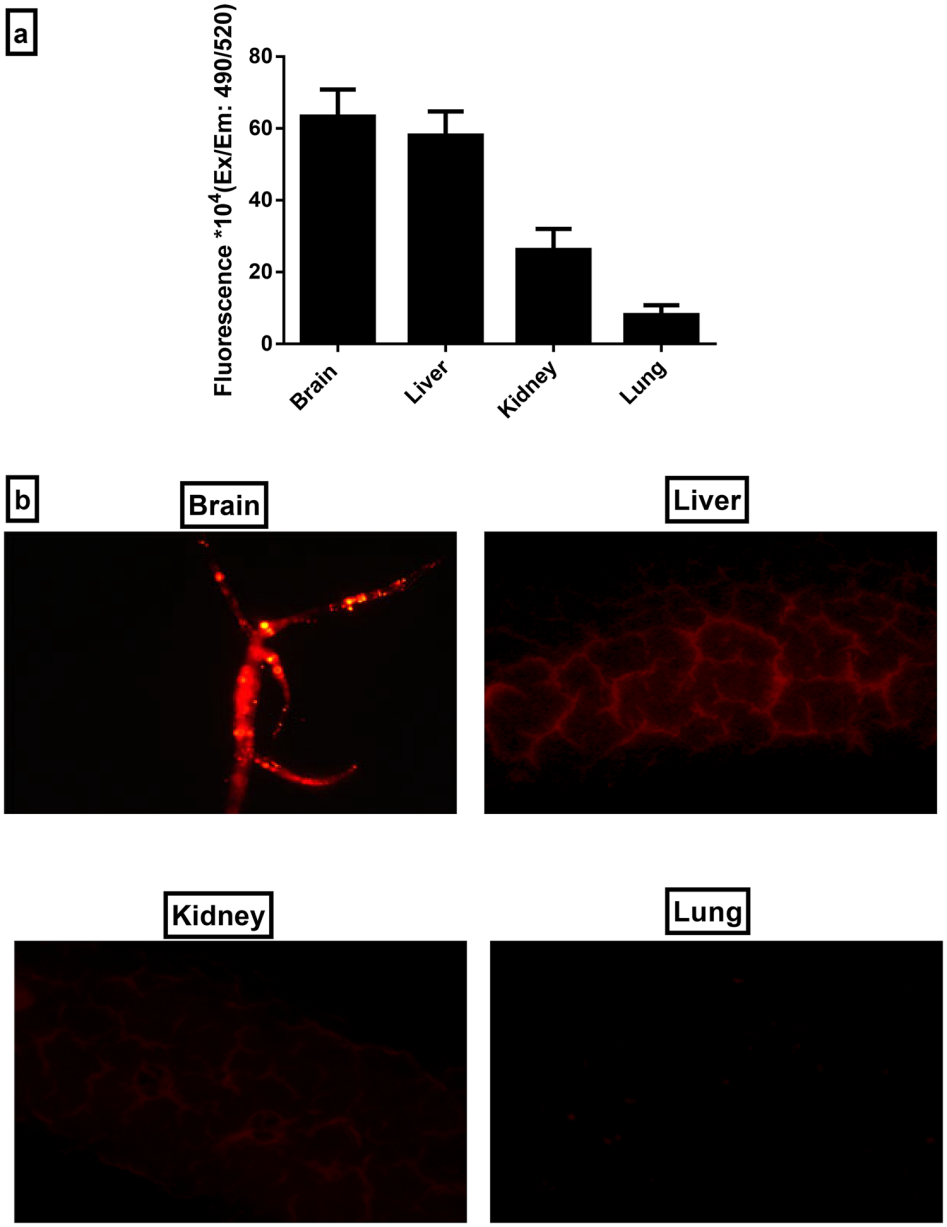
In vivo biodistribution of fluorescein diacetate-labeled ASH-BPNC in tissues of vital organs. Representative graph showing biodistribution of fluorescein diacetate-labeled ASH-BPNC in homogenized tissues of vital organs (**a**) and photomicrographs showing fluorescent imaging of tissue sections of vital organs treated with fluorescein diacetate-labeled ASH-BPNC (**b**). Data are represented as mean ± SD

The biodistribution of ASH-BPNC-FDA in the tissues of the examined vital organs (brain, liver, kidney, and lung) was first analyzed using a spectrofluorometer. Our results revealed that ASH-BPNC-FDA was extensively distributed in the brain and hepatic tissues, followed by the kidney and lung tissues (Fig. 13).

Similarly, the in vivo fluorescence imaging showed that the administration of ASH-BPNC-FDA demonstrated higher fluorescence signaling in both liver and brain tissues. Also, some fluorescence signaling was observed in kidney tissues. Minimal fluorescence signaling was observed in the lung tissues, which accounts for trivial accumulation (Fig. 13).

## Discussion

ASH is an essential herb in the Ayurvedic and indigenous medical systems. Its biologically active chemical constituents support its pharmacological significance as antioxidants, immunomodulatory, hypolipidemic, chemopreventive, anti-inflammatory, anxiolytic, and antidepressant agent [44]. However, ASH suffers from poor bioavailability because of the properties of its main bioactive compounds [23, 45]. Therefore, ASH-BPNC was designed to improve the physical stability of ASH in the GIT environment as well as its therapeutic efficacy.

In this study, the NE core was made of ASH and castor oil covered with Tween (with high hydrophilic–lipophilic balance) and Span (with low hydrophilic–lipophilic balance) to improve the loading of the extract and to stabilize the NE core. The core was then protected by a bipolymeric shell, in which a negatively charged polymer (alginate) coated the NE followed by ionic gelation with calcium chloride and chitosan to form a double-layer shell.

We investigated the effect of both polymers on particle size, zeta-potential, and ASH entrapment. Increasing the concentrations of both chitosan and alginate significantly increased the particle size. Conversely, increasing the alginate concentration decreased surface positivity, whereas increasing the chitosan concentration increased surface positivity. Chitosan/alginate mass ratios of 0.067:1 and 0.1:1 NC were previously studied [34], and these low chitosan ratios were found to result in a net NC charge between −19 and −23 mV, suggesting that the negatively charged polymer (alginate) dominated the net charge. Conversely, when chitosan/alginate mass ratios of 1:0.5 and 1:1 NC were examined, the net NC charge ranged from 40.6 to 45.5 mV [32]. These reports are in line with our current study, where the charge increased with increasing chitosan concentration from 25.2 to 37.2 mV at a low concentration of alginate and from 18.1 to 29.2 mV at a high concentration of alginate.

The EE% for all formulations was approximately 80%. The emulsification process and attributes of the materials controlled the ASH extract entrapment, thereby stabilizing the EE% across different formulations. This indicates that the developed NCs suitably encapsulated the ASH extract. The DSC thermogram further proved the successful assembly of the NCs, with significant peaks observed. Additionally, TEM imaging and dynamic light scattering showed similar sizing results, with a particle diameter of approximately 250 nm. The NC’s dissolution profiles fitted a Hixson–Crowell model, with fast release over 12 h (~60%) and followed by a slow release up to 48 h (~40%). On the basis of all these properties, we selected NC formula with 0.03% alginate and 0.03% chitosan to encapsulate ASH and investigated its therapeutic efficacy against TAA-induced HE.

HE is a recurrent neuropsychiatric complication of end-stage liver disease, which significantly affects the patients’ quality of life and increases the healthcare burden [46]. In preclinical studies, TAA has been extensively applied in experimental animals to induce hepatic failure with HE. It triggers hepatic and brain disorders resembling human HE pathophysiology, including motor incoordination, cognitive deficits, hyperammonemia, oxidative stress, and hepatic necrosis [47].

In the current study, TAA intoxication induced typical features of ALF, demonstrated by a pronounced elevation in ALT and AST serum levels, which indicates a loss of membrane integrity and subsequent leakiness in hepatocytes [48]. TAA intoxication also caused disrupted hepatic architecture as manifested by hepatocellular necrosis with marked parenchymal loss, mononuclear inflammatory cell infiltration, and fibrosis. The administration of 400 mg/kg ASH improved the distorted hepatic architecture and reduced the elevated levels of liver enzymes. However, better hepatoprotective effects were observed with 100 mg/kg ASH-BPNC, as indicated by restoration of ALT and AST levels and almost restoration of the TAA-induced disrupted hepatic architecture.

Hyperammonemia is considered one of the main contributing factors in the pathogenesis of HE. Under physiological conditions, ammonia is largely metabolized by the liver. However, in ALF, the hepatic clearance of ammonia is deficient because of a reduction in hepatic GS, a vital enzyme in ammonia homeostasis, causing an increase in systemic ammonia levels [49, 50]. Ammonia then crosses the blood–brain barrier to be detoxified in the brain by GS, which is mainly found in the astrocytes. This, in turn, results in the accumulation of glutamine in the astrocytes, causing cerebral edema and progression of HE [51]. Consistent with this, TAA intoxication in our study resulted in a marked increase in the systemic ammonia levels associated with a reduction in both hepatic and brain GS levels that caused astrogliosis, neuronal degeneration, and astrocyte swelling, leading to brain edema. Similar to our previous study [14], treatment with ASH reduced the elevated serum ammonia levels, as well as the hepatic and brain GS levels. Interestingly, incorporating ASH into BPNC further improved the systemic ammonia and hepatic and brain GS levels to the point of restoration. This was associated with amelioration of TAA-induced neuronal degeneration and astrocyte swelling.

Astrocytes play an essential role in the pathogenesis of HE; they regulate the blood–brain barrier and contain GFAP, which is responsible for maintaining their morphology and function [52, 53]. A previous study reported a reduction in the GFAP expression during ALF leading to a disturbance in the astrocyte function [54]. In the present study, TAA intoxication resulted in an increase in the immune expression of GFAP, which was alleviated by the administration of ASH or ASH-BPNC.

Oxidative stress notably contributes to the pathogenesis of ALF [55] and HE [18]. Overproduction of reactive oxygen species (ROS) causes lipid peroxidation, which leads to further damage to the cellular membrane and apoptosis induction [56]. This is accompanied by the depletion of endogenous antioxidant enzymes such as GSH [57]. Furthermore, oxidative stress downregulates Nrf2, leading to the inactivation of the antioxidant enzymes gene expression including HO-1 and GSH [18, 55]. Inactivation of Nrf2 pathway has been recorded in astrocytes [18], TAA-induced hepatic damage [58], acute liver failure [19, 59], and brain edema accompanied with HE [18]. Likewise, the present study showed that TAA intoxication resulted in increased ROS production in hepatic and brain tissues, as demonstrated by the increased MDA levels, the end product of lipid peroxidation, and the depletion of GSH stores. This was complemented by the suppression of the Nrf2 pathway, as indicated by the decline in Nrf2 and its target genes in liver and brain tissues [14]. ASH was previously shown to have powerful antioxidant effects in the TAA-induced HE model, which was mediated by the Nrf2 signaling pathway [14]. In the present study, ASH-BPNC notably restored the MDA and GSH levels and triggered translocation of Nrf2 from the cytoplasm to the nucleus, as demonstrated by the reduction in oxidative stress-induced brain edema and hepatic injury. This was further confirmed by the pronounced upregulation in the gene expression levels of Nrf2 and activation of its downstream ARE-target genes HO-1, GCLC and NQO-1 in hepatic and brain tissues. Our findings indicate that ASH-BPNC has cytoprotective effects, which is partly owed to activation of the Nrf2/ARE pathway in hepatic and brain tissues.

The interplay between Nrf2 and enhanced inflammation has been previously documented in HE, where an upregulation of Nrf2 correlated with a reduction in NF-κB transcription [14, 60]. In the present study, ASH incorporated into BPNC had enhanced both the antioxidant and anti-inflammatory activities compared with ASH treatment alone, as demonstrated by enhanced elevation in Nrf2 signaling and downregulation in the immunohistochemical expression of NF-κB in both hepatic and brain tissues.

Accumulating evidence demonstrates a significant contribution of peripheral inflammation to HE [61]. Peripheral inflammation has been reported to induce neuroinflammation, especially increased hippocampal NF-κB and TNF-α expression. This promotes the infiltration of blood cells, especially lymphocytes, into the brain, leading to microglial and astrocytic activation [62, 63]. Similarly, TAA intoxication caused a marked increase in hepatic inflammation, as denoted by an elevation in the immunohistochemical expression of NF-κB and TNF-α in hepatic tissues, suggesting an increase in peripheral inflammation. This was supplemented by an upregulation in the immunohistochemical expression of NF-κB and TNF-α in brain tissues. Notably, ASH-BPNC ameliorated TAA-induced inflammation in both hepatic and brain tissues.

In TAA-intoxicated rats, cognitive deficits, and motor incoordination accompanied by a reduction in the survival rate and neurological score have been previously reported [64]. TAA intoxication results in neuroinflammation leading to altered neurotransmission, which impairs spatial learning and causes motor incoordination [62, 63]. These effects were confirmed in our previous study [14]. In the current study, TAA-intoxicated rats displayed a reduction in motor activity and deterioration in cognitive functions as visualized by the decline in the number of crossing squares and rearing activity in the open field test. A reduction in learning indicators has been also observed, including the total exploration time, novel object preference percentage, and SAP. However, the administration of ASH or ASH-BPNC resulted in a marked increase in general motor activity. Interestingly, the administration of ASH-BPNC was able to improve the cognitive abilities of TAA-intoxicated rats and to restore their expected values better than ASH alone. This improvement was concomitant with a high survival rate and neurological score.

In addition to hyperammonemia, oxidative stress, and inflammation, MAPK family members, including p38 and ERK, contribute as well to the pathogenesis of HE. Oxidative stress and hyperammonemia are reported to trigger the activation of the MAPK pathway, resulting in NF-κB transcription and increased cytokine production in hepatic and brain tissues, thus leading to further HE progression [14, 65]. Our findings revealed that treatment of TAA-intoxicated rats with ASH-BPNC did not only restore serum ammonia levels, enhance antioxidant status, and inhibit hepatic and neuroinflammation, but also restored the gene expression levels of p38 and ERK1/2.

Our findings were further corroborated by the in vivo biodistribution studies, where most of the administered fluorescent-labeled NCs accumulated in the brain and liver tissues. We also observed some fluorescence in kidney tissues, which could be attributed to its function as an eliminating organ for NPs [66]. Moreover, renal failure is reported to be a major complication in patients with decompensated cirrhosis and is associated with high mortality and morbidity [67]. Since ASH has been demonstrated to possess renoprotective effects [68, 69], thereby the accumulation of some of ASH-BPNC in renal tissues could reflect an additional beneficial effect of ASH.

In our previous study, we reported the bioactivity of ASH against HE and investigated the chemical profile of ASH root aqueous extract using liquid chromatography–mass spectrometry. We identified > 45 compounds in ASH belonging to various metabolite classes, mainly flavonoids, phenolic acids, and steroidal lactone triterpenoids (including withaferin A, dihydrowithaferin A, dihydro-methoxy withaferin A, withanosides, and withanolides) [14].

## Conclusions

Our findings revealed that ASH extract was successfully loaded in chitosan–alginate bipolymeric NCs (BPNCs). In TAA-induced HE rat model, ASH-BPNC revealed enhanced therapeutic effectiveness compared with with ASH alone. This was evidenced by the lower adminstered dose, and higher survival rates, hepatoprotective and neuroprotective effects, as well as improvement in behavioral deficits. Moreover, the In vivo biodistribution studies demonstrated that most of the administered ASH-BPNC accumulated in liver and brain tissues. Our data denotes that BPNC acted as a successful oral delivery NC system for ASH, thereby providing a promising therapeutic approach for HE. Future pharmacokinetics/pharmacometabolomics–pharmacodynamics studies shall be conducted to investigate the synergism between the bioavailable compounds within the ASH extract. Further preclinical and clinical investigations are also warranted.

## Data Availability

The datasets generated during and/or analyzed during the current study are available from the corresponding author on reasonable request.

## Declarations

### Animal studies

All institutional and national guidelines for the care and use of laboratory animals were followed.
